# Navigating toward acceptance of death: Home-dwelling patients in the palliative phase

**DOI:** 10.1186/s12904-025-01707-4

**Published:** 2025-03-14

**Authors:** Katrine Staats, Sandra Jahr Svendsen, Veronica Lockertsen

**Affiliations:** 1 Faculty of Health Sciences, Department of Nursing and Health Promotion, Head of Studies - Area of responsibility 2, OsloMet– Oslo Metropolitan University, Kunnskapsveien 55, 2007 Kjeller, Norway; 2 Lillestrøm Municipality, Jonas Lies gate 18, 2000 Lillestrøm, Norway

**Keywords:** Home-dwelling patients, nurses, palliative care, acceptance, meaning, end-of-life care, death

## Abstract

**Background:**

As global life expectancy increases, the need for palliative care grows. Recognizing the deeply personal and diverse nature of individuals’ end-of-life experiences, palliative care for home-dwelling patients requires a flexible and person-centered approach. This study explores the complex process of death acceptance as experienced by patients receiving palliative care at home.

**Methods:**

Utilizing a qualitative, explorative, and descriptive design grounded in hermeneutic methodology, this study incorporates a secondary analysis of data derived from 13 in-depth interviews with home-dwelling patients in the palliative phase.

**Results:**

The findings reveal that patients consider relationships with family and healthcare professionals crucial in their journey toward accepting death. While strong, supportive relationships provided peace and meaningfulness, they could also introduce emotional complexity. Trust, honesty, and supportive care were fundamental for patients to find meaning and maintain quality of life during this challenging process. Staying in familiar environments, particularly at home, increased patients’ likelihood of accepting their mortality. Participants noted that achieving peace and reconciling with death required balancing hope for life with acceptance of death.

**Conclusion:**

This study highlights the complex process of death acceptance as experienced by patients receiving palliative care at home. Healthcare professionals can provide vital support by facilitating open conversations about fears and preferences related to death. Further research is needed to explore how end-of-life care can best support this intricate process.

## Introduction

Globally, life expectancy is on an upward trend [1]. While this signifies advancements in healthcare and living condition, it also leads to an aging population with a higher incidence of chronic and terminal illnesses. Consequently, the adult mortality rate is expected to rise, placing an enormous burden on society and healthcare systems [1, 2]. By 2060, it is estimated that nearly half of all people facing death worldwide will experience significant health-related suffering, underscoring the importance of prioritizing symptom alleviation and palliative care [2]. Palliative care is an approach aimed at improving the quality of life for people living with incurable illnesses [3]. It plays a crucial role in fostering acceptance of death and peaceful dying [4]. Acceptance in itself, is a complex concept intertwined with how patients cope with their illness and prognosis [5]. According to Kubler-Ross, death acceptance is characterized by a sense of peace and understanding about the inevitability of death. It is not about giving up or feeling defeated; rather, it is an adaptive response wherein the individual acknowledges the reality of the situation and begins to make peace with it [6].

This approach requires a holistic mindset, including physical, psychosocial, and existential care, due to the multidimensional nature of humans [4]. As the disease progresses, the needs of people in the palliative phase will increase, requiring more comprehensive and flexible treatment and care from healthcare professionals (HCPs) [7, 8]. It is crucial to recognize that the journey toward the end-of-life is deeply personal, characterized by a diversity of individual preferences [9]. Many prefer to remain in familiar surroundings, maintaining their daily routines and autonomy [10, 11]. The home environment often provides the safest and most satisfying setting for patients, enhancing family time and potentially facilitating acceptance of mortality [12]. However, home settings can also be stressful for families and caregivers, who may face significant emotional distress and loneliness in their roles [13]. These stressors can impact the caregivers’ own ability to cope and find acceptance of the impending loss. Therefore, acknowledging these complexities ensures a more comprehensive understanding of the palliative care experience in home settings. As acceptance can manifest in various ways, from an active search for acceptance to reluctant imposition of acceptance [12], the context of specialized services such as hospice care, seem to foster a higher level of acceptance for patients nearing end-of-life. This suggest that the nature of care received, as well as the place og care, can significantly impact how patients come to terms with their imminent death [5].

According to Norwegian parliamentary reports, health and care services should be based on individual values and desires, addressing physical, psychological, social, spiritual, and existential needs [7, 14]. This comprehensive approach can enhance awareness about death and encourage existential conversations, potentially helping individuals cope better with end-of-life challenges and reach acceptance [7]. Building on this, nurses play a crucial role in developing strategies and providing support to help patients navigate toward acceptance of death, enabling them to live their remaining time peacefully [15, 16].

The concept of a “good death” often emerges in discussions among stakeholders and HCPs when talking about death acceptance [17]. A “good death” is defined as one in which the patient and family recognize impending death and have a readiness and acceptance towards it [18]. According to Zimmermann [19] acceptance is a critical element in achieving a “good death”, also aligning with the goal of care in palliative settings. Moreover, society increasingly expects both patients and healthcare providers to embrace acceptance as part of the dying process, viewing it as a necessary step towards emotional and psychological peace. This societal narrative may, however, influence how individuals accept their imminent death and create unrealistic expectations [17]. “The Lancet Commission on the Value of Death” [20] illustrate this by suggesting that death and dying have become unfamiliar concepts, making it difficult to accept their inevitability. However, open acceptance of dying may not be appropriate for everyone in the palliative stage [21]. Therefore, it is crucial to acknowledge that each person’s journey toward the end-of-life is unique when navigating the acceptance of death. Additionally, the Commission states that death and dying are complex, multi-dimensional concepts deeply influenced by cultural contexts. This diversity further underscores the wide range of individual preferences in accepting death.

People’s interpretations of death can vary and evolve over time, shaped by social interactions and symbolic communication with others [22]. This underlines the importance of listening to patients’ voices in discussions about what constitutes a “good death” and how they navigate toward acceptance. Previous research, although rarely involving home-dwelling patients, acknowledges that the journey toward acceptance and a “good” death as encompasses physical, psychological, social, and spiritual dimensions [17, 23]. Accordingly, this study aims to explore the complex process of accepting death experienced by home-dwelling patients in the palliative phase. A better understanding of how patients come to terms with their mortality can encourage HCPs to provide more emphatic and person-centered palliative care at home.

## Methods

### Research design

This study employed an explorative, descriptive, and qualitative approach rooted in hermeneutic methodology. A secondary analysis was conducted using previously collected data from a primary study, as the original data contained rich, yet unexplored information that could answer new research questions [24, 25]. In the primary study, the second author interviewed 13 home-dwelling patients in the palliative phase to investigate their experiences with shared decision-making (SDM) and their preferences for engagement in such processes [26]. The participants also shared extensive experiences about their current life situations, including what they found meaningful and what promoted acceptance of their imminent death. As this perspective was not explored in the primary study [27], we conducted a secondary analysis. In this secondary analysis, we aimed to explore how the same target group navigate towards an acceptance of death. This new question drove our analytical thinking and guided our approach throughout the study [28]. The re-use of data is particularly important when the target group is difficult to access and in a vulnerable life situation [24]. As researchers, we must consider ethical implications, minimizing the burden on individuals and reducing their frequency of participating in research [29]. Moreover, in secondary analysis, it is recommended to utilize analytical techniques that align closely with those used in the primary research. Therefore, to answer the new research questions, Gadamer’s hermeneutical methodology, emphasizing the hermeneutic circle, was used to interpret the transcribed text [30]. This approach enabled us to develop a deeper understanding of the text’s meaning while continuously refining our pre-understanding.

To provide context for our secondary analysis, we first offer an overview of the primary study’s methodology [26].

### Participants and data collection of the primary study

The second author contacted six Norwegian municipalities, where cancer coordinators and nurses in the healthcare services assisted with recruitment. A purposive sample of 13 home-dwelling patients was interviewed between November 2022 and May 2023. The inclusion criteria were as follows: diagnosis of a life-limiting illness in the palliative phase, age over 18 years, capacity to provide informed consent, and willingness to share their experiences for research purposes. The participants were between 55 and 94 years old, with an average of 75 years. Most had cancer diagnoses, except for one who had COPD (chronic obstructive pulmonary disease). There were variations in terms of gender and housing as well as the degree of support provided by HCPs (see Table 1).

**Table 1:** Study participants. Socio-demographic data

(*N*=13)	
**Sex**	
Female	8
Male	5
**Age**	
<60 Years	3
61-70 Years	0
71–80 Years	5
81–90 Years	3
91–100 Years	2
**Housing**	
Living with spouse	5
Living alone	8
Urban	8
Rural	5
Community heath support*	
GP and HCS	13
CC	12
PCT	6
**Diagnosis**	
Malignant	12
Non-Malignant disease	1

*GP = General Practitioner, HCS = Home Care Services, CC = Cancer Coordinator, PCT = Palliative Care Team. Table adapted from Svendsen et al. (2024) [23]

*GP = General Practitioner, HCS = Home Care Services, CC = Cancer Coordinator, PCT = Palliative Care Team. Table adapted from Svendsen et al. (2024) [23].

The second author conducted individual, in-depth interviews in patients’ homes, creating a comfortable environment for open sharing of thoughts and experiences. Interviews lasted between 38 and 73 min. A modifiable semi-structured interview guide was used to explore emerging themes, in line with the hermeneutical methodology, which encourages new understandings through an ongoing interaction between the understanding of the whole and its parts, and vice versa [30]. Examples of questions leading the interview were: *Can you say something about what feels meaningful and important to you in your current situation?* and *In what way do you want others to be involved in your thoughts and decisions concerning the end-of-life?* All interviews were recorded and transcribed verbatim.

### Second analysis using the hermeneutical circle

To gain a comprehensive overview of the data, the first and last authors initiated the circular hermeneutical process by repeatedly reading and re-reading the uncoded transcripts [31]. This secondary analysis was undertaken with a new research question, distinctly different from the primary analysis, leading to the creation of new themes. We treated the data as a new data set and worked closely with the second author who was integral with the primary study, ensuring that all movements in the hermeneutical circle were checked and validated with her. Preliminary notes were recorded in a shared document and discussed with the second author throughout the interpretation process. Following Gadamer’s hermeneutical approach [30], the team moved between their preliminary understanding, aspects of the transcribed text, and initial interpretations of the text as a whole. We remained conscious of each researcher’s preunderstanding and its potential impact on the study’s results. Finally, we developed themes and sub-themes to represent our final interpretive understanding, ensuring a rigorous and thorough interpretation of the data in the context of our new research question.

### Preunderstanding

As researchers, our preunderstanding was not neutral or detached but characterized by a committed relationship to the subject. All authors are registered nurses with varied work and research experiences related to palliative care and existential matters. To ensure transparency and trustworthiness, we discussed and challenged our preunderstanding [32]. Our research team consisted of an associate professor with formal and clinical expertise in palliative care, a PhD candidate with substantial clinical experience in the same domain, and a mental health nurse with both clinical and research expertise related to existential questions for patients living in vulnerable life situations. Our preunderstanding was grounded in the assumption that patients nearing end of live derived comfort and a sense of meaningfulness from being in familiar surroundings. We also assumed that the participants’ acceptance of death was closely linked to their level of suffering, which heightened awareness of impending death. However, during the hermeneutical interpretation phase, the participants’ perceptions challenged our initial preconceptions, leading to a new understanding and a deeper meaning of the subject.

## Results

Data interpretation revealed that home-dwelling patients in the palliative phase experienced a multifaceted journey toward accepting death. This journey can be described in three themes: *The dynamics of relational interactions in end-of-life care*; *The importance of home and everyday normality*, and *Emotional and existential reconciliation.*

### The dynamics of relational interactions in end-of-life care

The participants’ relationships with family, friends, and HCPs played a central role in their journey toward accepting life’s end. Strong and supportive relationships helped create a sense of peace, reconciliation, and meaningfulness. However, these relationships not only added extra meaning to the final phase of life but also make it harder to approach thoughts of dying. Participants expressed how difficult it was to “die from” and miss out on the future of those close to them. During their precious remaining time, participants often adapted their interactions to what they could manage, shifting their focus from a larger circle of acquaintances to an inner core of the most significant people in their lives. Sharing and being close to loved ones was crucial for their sense of meaning during this period.

*Spending as much time as possible with my daughter is the most important thing in the world now. The hardest part of all this (cries) is that I’m going to pass away and leave her behind. That’s the only thing that matters. And she bakes the world’s best cinnamon rolls (alternates between crying and laughing) which we keep stuffing ourselves with.* (Woman, 55 years old)

Close relationships can also be challenging in this situation. In an attempt to spare their children, some participants tried to withhold information about the severity of their health condition. They felt their situation would be such a large burden on their children that they would rather bear it alone. However, the children viewed this as a breach of trust, which led to grief for both parties. In addition, these circumstances made it more challenging for both relatives and patients to accept the situation and feel supported emotionally during this last period of life. This suggests that in such a vulnerable period, both the environment and the person who is dying need to be trustworthy.

*I wasn’t honest with my children. It was in the early summer when they were dealing with exams*,* so I chose not to tell them that I was going for radiation therapy. That was not a good decision. It has led them not to trust me afterwards. So*,* you learn*,* but… (cries). It’s just that you try to protect. No*,* there’s something about being honest about everything and open about everything.* (Man, 58 years old)

The goal for some participants was to maximize the number of good days spent with their loved ones. This required a clear recognition of their reality, ensuring that neither they nor their family members clung to unrealistic expectations. For others, the thought of death elicited such severe anxiety that it became a topic too distressing to consider, and therefore, the theme of acceptance was never actualized. Many participants needed to process their thoughts and feelings verbally and thus turned to HCPs for support. Cancer coordinators and homecare nurses, perceived as compassionate, provided the necessary time and attention for participants to share their thoughts and feelings about the uncertainties of death:

*So the nurse sat here with me for about twenty minutes*,* and I cried and carried on (…) I have a daughter who can’t cope with my being unwell and just slams the doors and leaves*,* which brings me down. And this becomes a source of distress for me.* (Woman, 73 years old)

Conflicts in close relationships can be particularly distressing during this phase. Participants reported dealing with children whose anger and inappropriate behavior disrupted their process toward acceptance of death. It was challenging to have relatives who could not empathize and were more concerned with themselves. In such cases, the participants needed to shield themselves from these conflicts to maintain positivity on good days. Some felt it was crucial to communicate clearly that they were dying and could not handle additional demands. They believed they had done their part to secure their loved ones’ futures and now needed to be allowed to die without extra stress.

*Now I can’t do more for my children and grandchildren. They have to fend for themselves*,* and I have to be able to decide for myself (…) I say*,* please let me be allowed to die now. There is no meaning left. I have done what I could. And I haven’t been a super mother or grandmother*,* or human for that matter*,* but I have done what I could to make things easier for them*,* so that they will have an easier time than I did.* (Woman, 81 years old)

### The importance of home and everyday normality

Participants agreed that being in their home environment, where they could maintain daily routines and be close to loved ones, enhanced their sense of normalcy and quality of life. The home provided them with a safe, familiar, calming, and supportive context. They felt the ability to live at home was essential in their situation, as everyday normality offered meaning and gave them the strength to accept their fate. It was easier to maintain a sense of self and personal identity at home. Simply sitting in a chair and watching life outside allowed them to stay connected to the world:

*We have renovated here*,* and I got such large windows. It is very pleasant to sit in this room*,* as I have a very comfortable chair to sit in. And those directly across there have a dog and the neighbor there has a dog too. So*,* some of the highlights are that I see there is life outside in the garden*,* yes…. Now it’s meaningful that time passes.* (Man, 94 years old)

Several participants were initially frightened to return home from the hospital due to concerns about receiving adequate help. However, they unanimously agreed that living at home increased the value of their remaining time. Trust in their caregivers was crucial for feeling secure and finding meaning in living at home. Good caregivers allowed them to focus on what was important without stressing about receiving support, helping them accept their situation more fully. Participants perceived a significant difference in their quality of life at home compared to being in a hospital or nursing home, where their sense of purpose and dignity diminished. At home, they could be themselves and maintain stability and dignity, even while receiving assistance.

### Emotional and existential reconciliation

The participants indicated that achieving peace and reconciling with death required them to balance between hope for life and acceptance of death. This balance was often influenced by various external elements, such as medical appointments set in the future. This significantly affected their perception of their limited future prospects. Participants clinging to life felt that these appointments reaffirmed their ongoing existence and served as a source of hope. In contrast, those who had accepted the inevitability of death found these appointments stressful and disruptive to their peace-making process.

*I do not wish to be admitted to the hospital unless they believe they can do something to improve my condition. I have no interest in being there merely for the sake of it*,* or for undergoing a MRI or a similar procedure. I am well aware of my situation*,* and I do not want to spend any of the time I have left being transported to and staying in the hospital. The cancer coordinator actually cancelled*,* I believe*,* five appointments. I had numerous appointments*,* even with the ophthalmologist…. Simply thinking about having to go makes me feel exhausted*,* empty even….* (Woman, 55 years old)

The intensity of the participants’ suffering was intricately connected to their expectations of the future. High levels of suffering made it difficult for them to envision a prolonged life. Ironically, the anticipation of ongoing discomfort often facilitated acceptance of death. As the end-of-life drew nearer, many participants reported increased clarity both mentally and emotionally. It seemed as though their bodies were gradually shutting down, naturally preparing their minds and spirits for the end. However, this process left them exhausted, feeling more like mere existence than living.

*I know I’m ill*,* but it’s as though I move in and out of this reality. It’s a process. Some days*,* I’m thoroughly tired of discussing cancer*,* other days it’s necessary to talk about it. (….) Looking after our mental health is important*,* as it can quickly turn into a mental disorder.* (Woman, 56 years old)

The participants’ journey toward accepting death involved various emotional and existential reflections. For some, this process included managing a spectrum of emotions, ranging from grief to relief or a sense of peace with their circumstances, with death transitioning from an existential dilemma to a simple, undeniable reality. Their doctor’s encouragement to stay positive while simultaneously viewing death as something negative complicated their emotional journey. It felt as though they were being pushed to be fearful of death, despite it being a natural part of life.

*The life we have is given to us*,* and in principle*,* it’s the only possession we truly own. I love living*,* but the question of death no longer feels existential to me. I’ve noticed that society expects me*,* as a patient*,* to be very fearful of death*,* which I’m not. They assume everyone is anxious.* (Man, 91 years old)

Several participants expressed satisfaction with their lives, reflecting back on many good years. While old age was frequently associated with this acceptance, not all felt that age alone prepared them for death. Some hoped for resuscitation if needed, although few had discussed such preferences with their physicians. Several of the younger participants spoke about feeling robbed of their rightful futures, making it harder to accept death. They felt as if they were in survival mode, striving to preserve everything that defined their identity and uniqueness, both physically and spiritually.

## Discussion

The results are discussed by focusing on the interaction around the “Acceptance of death,” highlighting the differences in how home-dwelling patients in the palliative phase experience the journey toward accepting death. Each outer circle will be explored through a dialogue that interweaves our empirical findings with existing research. Additionally, each circle will be discussed based on the theoretical framework of symbolic interactionism.

### Relational dynamics in the dying process

In our study, we found that participants’ relationships with family, friends and HCPs played a central role in their journey towards accepting death. Relatives are crucial supporters for many home-dwelling patients in the palliative phase. However, patients have different needs for connection, closeness, and sharing of their process. For some, the tendency to distance themselves from others was connected to difficulties in coping with their own situation. As patients faced impending death, only their closest relatives became part of their “inner circle.” In accordance with established theoretical knowledge [33], this inward shift of focus, away from interaction with people in one’s environment, is a normal part of the journey toward death. It allows patients to prepare mentally and emotionally for their ultimate transition.

Conversely, many patients emphasized the importance of involving their relatives in small, everyday activities to share as much as possible while they can. They tended to share information and the dying process with these relatives, as if they collectively bore the responsibility for navigating this final journey. Daneault et al. [34] also highlighted the impact of this crucial familial contact, arguing that it is not what close family members do but rather their mere presence that brings comfort. Similarly, Prado et al. [12] noted that patients nearing end-of-life prioritize family life, seeking protection and support. However, they also found that patients and their family members accept this inevitable process in different ways, particularly when the reality of imminent death is unavoidable.

These relational challenges can be discussed in light of the framework of symbolic interactionism [22], which explains how people form meanings for their actions and social relations. As described by Blumer [22], interactions are essential for individuals to interpret and respond to their reality. Being conscious and aware of how people recognize and define their own actions and expressions, as well as those of others, is crucial in this transitional phase for home-dwelling palliative patients. As such, acknowledging that the path toward the end-of-life is highly individual, marked by a wide range of personal choices and preferences—both for the patient and their family members—is important [9]. This relates to our findings, highlighting how various relational circumstances can disrupt the process of acceptance. While some patients had long-term conflicts with close relatives, limiting supportive interactions, others experienced relationship disruptions during their illness, often due to efforts to protect family members from practical and emotional burdens. Nevertheless, being aware of the patients’ relationships and how they offer the patient support and are prepared to listen is an important factor in palliative care, as openness about emotional reactions is a vital part of patients’ possible acceptance of death [19].

In our study, withholding information during a vulnerable period created a breach of trust within relationships. When interactions involve conflict and a lack of communication, it may be challenging to construct positive meanings around the end-of-life experience because the conflicts disrupt supportive interactions, which are crucial for mutual understanding [35]. Thus, difficult relationships can hinder the patients’ ability to exploit the potential space for acceptance. The dying often attempts to accept and move toward the end, while their relatives prepare for a life without them. This dynamic is influenced by anticipatory grief, where family members focus on the anticipated losses after the person’s death, adding complexity to their interactions and emotional responses [36]. Here, trust in HCPs is crucial, as they can help to navigate difficult relationships [15]. HCPs have the opportunity to develop person-centered strategies that support patients in accepting death and facilitating their remaining time peacefully with family [37]. Curiosity about what the individual considers a “good death”, and the meaning of this last period is crucial, emphasizing the importance of the symbolic environment in their acceptance process [17].

### The symbolic significance of feeling home when nearing end-of-life

In our study, patients reported that being in a home environment where they could maintain daily routines and stay close to loved ones enhanced their sense of normalcy and quality of life. The familiarity and comfort of home were calming and supportive, facilitating their process of accepting death. According to symbolic interactionism, by continuously interacting with familiar belongings collected throughout their lives, patients can connect and manifest their identity through these environmental features, helping them feel more like themselves rather than just dying individuals [35]. Maintaining everyday life affirms their value as individuals and makes their remaining time more meaningful. Indeed, Nysæter et al. [11] found that next to staying in one’s home, living a meaningful life to the extent possible prior to death is crucial. This highlights an important dichotomy. While the physical home may hold symbolic value, and any disruption to this environment may complicate the acceptance of impending death, the feeling of being at “home” can also be created elsewhere [10]. Staats et al. argued that nurses’ attitudes and action of care are important and should be individualized for patients and their families in this phase of life, irrespective of the context [10]. Zimmerman [19] underlines how caregivers, by explaining, teaching and preparing, facilitate how both families and the patients cope with and accept the terminal prognosis.

Overall, our findings enrich the understanding of how the place of residence influences the engagement of palliative patients by trusted caregivers. This interaction allows patients to focus on what is important and helps them more fully accept their situation. By highlighting the profound impact of home and everyday normalcy, our study contributes to the sparse base of knowledge concerning how dying home-dwelling patients in the palliative phase can face death with a greater sense of peace and understanding.

### Societal paradoxes and the emotional acceptance of dying

We found that the emotional and existential acceptance of death and dying is closely related to societal viewpoints. Overtreatment and society’s view on death create a paradox, as death is seen as natural but also as something to be postponed. The patients in this study experienced being scheduled for unrealistic future magnetic resonance imaging (MRI) appointments, which interfered with their acceptance process. The symbolic action of scheduling future appointments evokes emotional reactions that could either support or undermine the patient’s emotional and existential reconciliation with their impending death [35], depending on their state of mind and acceptance process. Patients who see continued treatment as hopeful interpret these actions as symbols of ongoing care and life. In contrast, those who find these appointments stressful interpret them as an unnecessary prolongation of suffering [35]. In either case, the symbolic meaning of these actions interferes with patients’ ability to accept their terminal condition because they send mixed messages about their prognosis [22]. When health care services deny or do not acknowledge the patient’s impending death, it becomes difficult for the patients to comes to term with their situation. Sallnow et al. [20] emphasized that the culture of overtreatment creates an imbalanced relationship with death. Treatment provided in the last months of life and avoidance of conversations about death and dying can cause patients and their families to struggle to accept the inevitability of death [20]. Similarly, Bosco and Cappellato [37] highlighted societal challenges in accepting death and dying. They found that measures for managing severe end-of-life symptoms are far less common than invasive procedures and treatments like chemotherapy for dying patients. Helping patients and families accept death presupposes a healthcare service open to a self-reflective attitude and a mindset that aligns with the ethos of palliative care [19]. However, it appears that the inevitability of death as part of the human condition is not sufficient to make it acceptable [37]. In summary, our findings highlight the intricate interplay of relationships, home environment, and emotional and existential factors in shaping the end-of-life journey and the process of accepting death.

### Strengths and limitations

Among the strengths of this study include the reuse of data. This secondary analysis yielded important qualitative findings regarding a sensitive topic in healthcare [24]. Reusing data on such a sensitive subject allowed for deeper insights without imposing additional burdens on participants. This approach maximizes the utility of existing data and promotes resources efficiency [28]. However, it is important to acknowledge how limited flexibility to ask follow-up questions and lack of the possibility to probe emerging themes were limitations of this secondary analysis and may have impacted the depth of the data [28]. Despite these limitations, the secondary analysis provided a robust platform to explore and validate a complex subject. The primary researcher in this study (second author) collected the data, and both the first and second authors participated in the primary study’s analysis. While the secondary researcher (last author) may not have had the same level of access to nuanced information, the primary researcher’s insights and detailed knowledge were integral to the analysis phase [25]. Also, all authors held regular meetings to bridge any gaps in this study context. This ensured that the author’s preunderstanding was transparent and consciously considered throughout the data analysis.

## Conclusion

The complex interplay of relationships and environmental elements is central to how home-dwelling patients in the palliative phase navigate their thoughts and views toward an acceptance of death. Also, this journey is both emotional and existential as achieving peace and reconciling with death required the patients in this study to find a balance between maintaining hope for life and accepting death. Trust, honesty, and supportive care are fundamental for finding meaning and maintaining quality of life during this challenging process. By addressing emotional conflicts, HCPs can help patients balance hope and acceptance through honest conversations about their fears and preferences regarding death and dying. Developing and maintaining strong, supportive relationships between patients, families, and HCPs can support this process. It is particularly crucial to understand the multifaceted nature of the acceptance process, which involves complex individual preferences and ideas. Further research is needed to understand how end-of-life care can best support this process. For example, studies could examine the perspectives of healthcare professionals and informal caregivers on this subject.

## Data Availability

Availability of the raw data is available from the corresponding author upon request.

## Abbreviations

HCP: Healthcare professional

## References

[1] World Health Organization. Global Health Estimates: Life Expectancy and Leading Causes of Death and Disability; Accessed July 10. 2024. https://www.who.int/data/gho/data/themes/mortality-and-global-health-estimates

[2] Sleeman KE, De Brito M, Etkind S, et al. The escalating global burden of serious health-related suffering: projections to 2060 by world regions, age groups, and health conditions. Lancet Glob Health. 2019;7(7):e883–92. 31129125 10.1016/S2214-109X(19)30172-X PMC6560023

[3] World Health Organization, WHO Definition of Palliative Care; Accessed July 17. 2021. https://www.who.int/teams/integrated-health-services/clinical-services-and-systems/palliative-care

[4] International Association for Hospice and Palliative Care (IAHPC). What is palliative care? Accessed July 17, 2024. https://hospicecare.com/what-we-do/publications/getting-started/what-is-palliative-care/

[5] Coast J, et al. Adaptation, acceptance and adaptive preferences in health and capability well-being measurement amongst those approaching end of life. Patient. 2018;11(5):539–46. 29744765 10.1007/s40271-018-0310-z

[6] Kübler-Ross E. On death and dying: what the dying have to teach Doctors, nurses, clergy and their own families. Oxon, Routledge; 2008.

[7] Ministry of Health and Care Services. No. 24 (2019–2020) to the Storting– palliative care and treatment– some day we will all die. But on all the other days, we will not. The Norwegian Ministry of Health and Care Services. Oslo; 2020.

[8] Carrasco-Zafra MI, Gómez-García R, Ocaña-Riola R, Martín-Roselló ML, Blanco-Reina E. Level of palliative care complexity in advanced cancer patients: a multinomial logistic analysis. J Clin Med. 2020;9(6):1960. 10.3390/jcm9061960. 32585859 10.3390/jcm9061960 PMC7356562

[9] Kyota A, Kanda K. The perception of life and death in patients with end-of-life stage cancer: A systematic review of qualitative research. Eur J Oncol Nurs. 2023;66:102354. 37586291 10.1016/j.ejon.2023.102354

[10] Staats K, Ervik B, Fæø SE. The feeling of being home when nearing end-of-life—the example of Norway: a discussion paper. Nord J Nurs Res. 2023;43(2):20571585231180185.

[11] Nysæter TM, Olsson C, Sandsdalen T, Wilde-Larsson B, Hov R, Larsson M. Preferences for home care to enable home death among adult patients with cancer in late palliative phase– a grounded theory study. BMC Palliat Care. 2022;21(1):49. 35410199 10.1186/s12904-022-00939-y PMC9004171

[12] Prado E, et al. Meanings and experiences of end-of-life patients and their family caregivers in hospital-to-home transitions: a constructivist grounded theory study. Int J Environ Res Public Health. 2022;19(20):12987. 36293568 10.3390/ijerph192012987 PMC9602127

[13] Staats K et al. End-of-life care at home: dignity of family caregivers. Nurs Ethics. 2024; pages: Pages 9697330241241773. 10.1177/09697330241241773 PMC11800689 38587469

[14] Ministry of Health and Care Services. No. 24 (2022–2023) community and Coping — Live safely at home. The Norwegian Ministry of Health and Care Services. Oslo; 2023.

[15] Upasen R, Thanasilp S. Death acceptance from a Thai Buddhist perspective: a qualitative study. Eur J Oncol Nurs. 2020;49:101833. 33120215 10.1016/j.ejon.2020.101833

[16] McCormack B, McCance T, Bulley C, Brown D, McMillan A, Martin S. Fundamentals of Person-Centred healthcare practice. Hoboken, NJ: Wiley; 2021.

[17] Krikorian A, Maldonado C, Pastrana T. Patient’s perspectives on the notion of a good death: a systematic review of the literature. J Pain Symptom Manag. 2020;59(1):152–64. 10.1016/j.jpainsymman.2019.07.033 31404643

[18] Cottrell L. Duggleby. The good death: an integrative literature review. Palliat Support Care. 2016;14(6):686–712. 26732508 10.1017/S1478951515001285

[19] Zimmermann C. Acceptance of dying: A discourse analysis of palliative care literature. Soc Sci Med. 2012;75(1):217–24. 22513246 10.1016/j.socscimed.2012.02.047

[20] Sallnow L, et al. Report of the lancet commission on the value of death: bringing death back into life. Lancet. 2022;399(10327):837–84. 35114146 10.1016/S0140-6736(21)02314-X PMC8803389

[21] Hilário AP. Preferences around the disclosure of dying: a vision from Portuguese society. Palliat Support Care. 2020;18(1):63–8. 31138342 10.1017/S1478951519000324

[22] Morrione TJ, Herbert, Blumer. Symbolic interactionism, and 21st-Century sociology. London: Routledge; 2021.

[23] Heimerl K, et al. Dying is never beautiful, but there are beautiful moments: qualitative interviews with those affected on the subject of ‘good dying’. Mortality. 2023;28(4):543–61.

[24] Long-Sutehall T, Sque M, Addington-Hall J. Secondary analysis of qualitative data: a valuable method for exploring sensitive issues with an elusive population? J Nurs Res. 2011;16(4):335–44.

[25] Chatfield S. Recommendations for secondary analysis of qualitative data. Qual Rep. 202;25:833–42.

[26] Svendsen SJ, Grov EK, Staats K. Patients’ experiences with shared decision-making in home-based palliative care– navigation through major life decisions. BMC Palliat Care. 2024;23(1):101. 38627710 10.1186/s12904-024-01434-2 PMC11022472

[27] Ruggiano N, Perry TE. Conducting secondary analysis of qualitative data: should we, can we, and how? Qual Soc Work. 2019;18(1):81–97. 30906228 10.1177/1473325017700701 PMC6428200

[28] Beck CT. Secondary qualitative data analysis in the health and social sciences. Oxford, Oxford: Routledge; 2019.

[29] Conway E, MacEachen E, Middleton L, McAiney C. Use of adapted or modified methods with people with dementia in research: a scoping review. Dementia. 2023;22(8):1994–2023. 37871184 10.1177/14713012231205610 PMC10644684

[30] Gadamer HG. Truth and Method. 2nd rev. ed. Weinsheimer J, Marshall DG, trans-eds. London: Sheed & Ward; 1989.

[31] Gilje N. Hermeneutikk Som Metode: Ein historisk Introduksjon. [Hermeneutics as a Method: A Historical Introduction]. Oslo: Samlaget; 2019.

[32] Lincoln YS, Guba EG. Naturalistic inquiry. Volume 75. Newbury Park, CA: SAGE; 1985.

[33] Oxford Textbook of Palliative Medicine. Cherny NI editors Oxford University Press; 2021.

[34] Daneault S, et al. Passing through end-of-life suffering: possible or not? Results from a qualitative inquiry. Death Stud. 2023;47(8):902–13. 36369723 10.1080/07481187.2022.2142326

[35] Powell JL. Symbolic interactionism. 1st ed. Hauppauge: Nova Science, Incorporated;; 2013.

[36] Singer J, et al. An examination and proposed definitions of family members’ grief prior to the death of individuals with a life-limiting illness: a systematic review. Palliat Med. 2022;36(4):581–608. 35196915 10.1177/02692163221074540 PMC10098140

[37] Bosco N, Cappellato V. Preparing for a good death? Palliative care representations in the Italian public television programming. Death Stud. 2022;46(8):1963–72. 33476248 10.1080/07481187.2021.1876788

